# Performance of Natural Language Processing versus International Classification of Diseases Codes in Building Registries for Patients With Fall Injury: Retrospective Analysis

**DOI:** 10.2196/66973

**Published:** 2025-07-14

**Authors:** Atta Taseh, Souri Sasanfar, Michelle Chan, Evan Sirls, Ara Nazarian, Kayhan Batmanghelich, Jonathan F Bean, Soheil Ashkani-Esfahani

**Affiliations:** 1 Foot & Ankle Research and Innovations Laboratory (FARIL), Department of Orthopaedic Surgery, Mass General Brigham, Harvard Medical School, 158 Boston Post Road, Weston, MA, 02493, United States, 1 7818279613; 2 Musculoskeletal Translational Innovation Initiative, Carl J. Shapiro Department of Orthopaedic Surgery, Beth Israel Deaconess Medical Center, Harvard Medical School, Boston, MA, United States; 3 Batman Laboratory, Department of Electrical and Computer Engineering, College of Engineering, Boston University, Boston, MA, United States; 4 New England Geriatric Research Education and Clinical Center (GRECC), Veterans Affair Boston Healthcare System, Boston, MA, United States; 5 Department of Physical Medicine and Rehabilitation, Harvard Medical School, Boston, MA, United States; 6 Spaulding Rehabilitation, Boston, MA, United States

**Keywords:** automation, automate, data registry, ICD Codes, artificial intelligence, AI, algorithms, predictive models, predictive analytics, machine learning, ML, large language models, LLMs, natural language processing, NLP, deep learning

## Abstract

**Background:**

Standardized registries, such as the *International Classification of Diseases* (*ICD*) codes, are commonly built using administrative codes assigned to patient encounters. However, patients with fall injury are often coded using subsequent injury codes, such as hip fractures. This necessitates manual screening to ensure the accuracy of data registries.

**Objective:**

This study aimed to automate the extraction of fall incidents and mechanisms using natural language processing (NLP) and compare this approach with the *ICD* method.

**Methods:**

Clinical notes for patients with fall-induced hip fractures were retrospectively reviewed by medical experts. Fall incidences were detected, annotated, and classified among patients who had a fall-induced hip fracture (case group). The control group included patients with hip fractures without any evidence of falls. NLP models were developed using the annotated notes of the study groups to fulfill two separate tasks: fall occurrence detection and fall mechanism classification. The performances of the models were compared using accuracy, sensitivity, specificity, positive predictive value, negative predictive value, *F*_1_-score, and area under the receiver operating characteristic curve.

**Results:**

A total of 1769 clinical notes were included in the final analysis for the fall occurrence task, and 783 clinical notes were analyzed for the fall mechanism classification task. The highest *F*_1_-score using NLP for fall occurrence was 0.97 (specificity=0.96; sensitivity=0.97), and for fall mechanism classification was 0.61 (specificity=0.56; sensitivity=0.62). Natural language processing could detect up to 98% of the fall occurrences and 65% of the fall mechanisms accurately, compared to 26% and 12%, respectively, by *ICD* codes.

**Conclusions:**

Our findings showed promising performance with higher accuracy of NLP algorithms compared to the conventional method for detecting fall occurrence and mechanism in developing disease registries using clinical notes. Our approach can be introduced to other registries that are based on large data and are in need of accurate annotation and classification.

## Introduction

With 3 million emergency room visits, 300,000 hospitalizations, and 30,000 fatalities annually, falls pose a major threat to public health [1,2]. The financial impact is also substantial, with an estimated US $50 billion medical expenses for nonfatal falls [3]. Therefore, researching and understanding the nature of falls and fall-related injuries are crucial for developing effective prevention and treatment strategies as populations age [4]. Given the multifactorial nature of falls and the difficulties involved in conducting prospective research in the field, developing fall registries comprised of large and accurate medical data is very important [5,6]. Standardized registries are commonly built using administrative codes, such as the *International Classification of Diseases* (*ICD*), assigned to patient encounters, and Current Procedural Terminology (CPT) codes[7]. Previous studies have used these codes to extract patients with a history of falls [8-10]. However, this method has limitations that may lead to an underestimation of actual fall frequency and might not reveal the history of falls in patients [11]. Reporting falls using the External Causes of Morbidity codes is usually recommended but not mandatory in all health care settings. Since falls are not typically considered stand-alone conditions, many health care providers may rather use the diagnosis *ICD* codes and assign codes to the end result of a fall, for example, a hip fracture, rather than the fall itself [12,13]. This makes it difficult for investigators to identify falls in the patient’s medical history and the true frequency of falls within populations. Given these limitations, clinical notes were suggested as a more reliable method of detecting falls, fall mechanisms, and fall-induced injuries [14]. This process, however, is expert-dependent and time-consuming, particularly if the dataset is large. To address these obstacles, natural language processing (NLP), which combines computational linguistics and deep learning models to process narrative data, can be used to automate the review process of clinical notes to detect falls [14].

Several studies have demonstrated the capability of supervised models to detect fall incidents, which have been documented in clinical notes [15-17]. Although these models are effective at identifying fall events, they fall short of providing detailed insights into fall-related *ICD* codes that capture the specific mechanisms (eg, how the fall occurred) or the physical consequences (eg, the force of the impact) [11]. Gaining a better understanding of these factors is essential for designing strategies to prevent falls since individuals who experience severe or high-impact falls often face a higher risk of recurrent falls and injuries [9,18]. Tremblay et al [11] highlighted the importance of studying fall mechanisms as a research priority. However, automated methods for extracting detailed fall mechanisms and their impact from clinical notes remain largely unexplored in the current literature.

This study aimed to assess the performance of NLP algorithms compared to conventional methods for detecting fall incidence and the mechanism of falls obtained from clinical notes of patients with hip fractures. We hypothesize that NLP algorithms outperform fall ICD codes in detecting falls and their mechanisms in patients with hip fractures.

## Methods

### Study Design and Cohort

A retrospective case-control study was conducted, including the data from 4 tertiary hospitals in Greater Boston, Massachusetts. Data were retrieved from the institution’s data repository using CPT codes for hip fractures (27125, 27130, 27226, 27228, 27235, 27236, 27244, 27245, and 27248) between January 2010 and December 2019.

Patients ≥18 years old who were hospitalized because of hip fracture as a result of an outpatient fall (cases) or other reasons (controls) were included in the study. Falls resulting from violent encounters, animal attacks, significant external forces such as car or motor vehicle accidents, high-impact sports like skiing, and fractures caused by underlying pathological conditions were excluded to reduce the heterogeneity of fall mechanics. This exclusion helps avoid the influence of confounding injuries that differ significantly from typical accidental falls, ensuring that the study focuses on more clinically relevant fall types (). Given that the majority of hip fractures happen due to falls, we had a reasonable number of patients in the case group and included a single note for each patient. In contrast, multiple notes were reviewed and included per patient in the control group.

### Data Labeling

Expert annotations, serving as the ground truth for training the NLP models, were derived directly from clinical notes. The annotations embraced two specific tasks: (1) fall occurrence and (2) mechanism of falls (the way falls happened). One expert orthopedic researcher (AT) conducted the annotations, and the decisions for equivocal or debatable cases were made by a senior scientist (SAE). All clinical notes were evaluated in chronological order, starting from the date of the hip fracture CPT code. The first note documenting a fall was selected for analysis. A fall was defined as “an unintentional event that results in the person coming to rest on the ground or another lower level” [19]. The mechanisms of fall were defined by 3 categories: same level (occurring on the same plane or surface), multilevel (descent from one level to a different one), and unclassified (not classifiable due to lack of sufficient information) [20]. In rare cases, discrepancies between the documented fall mechanisms in the clinical notes and the corresponding fall *ICD* codes compromised the validity of comparisons between *ICD* and NLP-based approaches. Consequently, patients with conflicting information between clinical notes and ICD codes regarding the fall mechanism were excluded to ensure the integrity of the analysis ().

### Data Preprocessing

A variety of inpatient unstructured clinical notes, including history and physical examination, discharge summary, progress, operation, and emergency department notes, were obtained. Due to the diverse formatting of these clinical notes, specialized preprocessing methodologies were required, which diverged significantly from the conventional text-processing approaches. Following annotation, the clinical notes underwent various preprocessing steps, including de-identification, segmentation, and cleaning [21]. The specific techniques used in preprocessing, which address the unique challenges posed by the clinical notes’ formatting, are outlined in Table 1. Detailed information about the segmentation process is provided in Tables S1 and S2 in Multimedia Appendix 1. This detailed account ensures the data are optimally prepared for the subsequent analytical phases.

**Table 1.** An overview of the data preprocessing stages.

Stages	Tool or Method	Purpose	Output
De-identification	Stanford de-identifier	Remove personal identifiers to ensure privacy and compliance with data protection regulations. This involves replacing all Protected Health Information entities with synthetic variants to maintain data integrity and eliminate biases. The model chosen was the Stanford-de-identifier-base-model developed by Chambon et al [21], with an *F*_1_-score of 98.9 on the I2b2 2014 test set [22].	Anonymized text ready for analysis.
Segmentation	Bespoke parser, Finite State Machine, and regular expressions	Segment notes into distinct sections for enhanced text processing accuracy. The parser identifies section headings and concatenates segments, refined through manual evaluation and iterative improvements. More details are provided in Multimedia Appendix 1.	Accurately segmented text with sections tagged for reassembly.
Filtering uninformative data	Identification and removal: Duplicates Uninformative sections Administrative content	Remove duplicated sections from notes to prevent skewing results. Discard sections containing only headings without informative text. Remove document finalization and signature sections marked with terms like “signed” and “FINAL.”	Dataset free of redundant and uninformative sections.
Elimination of non-essential elements	Regular expressions and manual filtering	Exclude conversion error notifications, Unicode or hexadecimal sections, and other irrelevant elements.	Dataset without non-contributory headers, unreadable sections, and irregular patterns.
Removal of irrelevant metadata	Manual filtering	Remove timestamps, de-identified placeholders, and other non-analytical metadata.	Dataset without timestamps and placeholder text, ensuring grammatical consistency.
Splitting the data	Random allocation	Partition the dataset into training and testing subsets for unbiased model evaluation.	Training and testing subsets for model development and performance evaluation.

### Model Development

Models were developed to automate two distinct tasks: fall occurrence and fall mechanism classification. All models besides Bidirectional Encoder Representations from Transformers (BERT) used a Term Frequency-Inverse Document Frequency (TF-IDF) representation of the text data. Specifically, TF-IDF vectorization with unigrams, bigrams, and trigrams (ngram_range=(1,3)) was applied to transform the processed text into numerical features before training these models. For the binary task of fall occurrence (fall vs no fall), a data split of 80:20 was used for training and testing purposes, respectively. The split was stratified by the binary outcome (fall vs no fall) to ensure a balanced representation of both classes in the training and testing subsets. Our methodology harnessed the text analysis capabilities of a modified BERT model described by Fu et al [17] We used a maximum sequence length of 512 tokens, consistent with the recommendations in the original study by Devlin et al [23], used a batch size of 8, and conducted training over 3 epochs. Moreover, the adaptive boosting (AdaBoost) algorithm was used for fall identification, using single-layer decision trees (stumps) as described by Quinlan et al [23], [24]. AdaBoost assigns coefficients based on each classifier’s performance and adjusts sample weights during training to emphasize previously misclassified samples. Finally, extreme gradient boosting (XGBoost) was used, which is a refined version of gradient boosting recognized for its precision and versatility. XGBoost constructs additive training models in stages and optimizes a differentiable loss function, making it suitable for handling structured data derived from text [25].

To address the challenges posed by the complex multiclass scenario in the fall mechanism classification task, which involved detailed classification into 3 categories (same level, multilevel, and unclassified classes), we designated 70% of the data for training and 30% for testing, ensuring stratification to maintain class distribution. We used a comprehensive suite of advanced machine learning models, including AdaBoost, support vector machine (SVM), XGBoost, and random forest (RF). Each model was chosen for its proven ability to decipher complex data relationships and offer detailed insights into the correlated factors of falls across the varied categories [26-31]. The SVM model is a two-layer recognition method that excels in high-dimensional spaces and allows for class weighting to address class imbalance, which makes it suitable for detecting fall mechanisms from clinical notes [32] RF is an ensemble learning method that constructs multiple decision trees during training and merges their results to improve predictive accuracy and control overfitting. RF is also effective in handling class imbalance through class weighting [33].

The hyperparameter configurations used for the models are provided in Table S3 in Multimedia Appendix 1.

### Statistical Analysis

Comparison of the baseline characteristics was made using SPSS software (version 28.0; IBM Corp), where the *t* and chi-square tests were used for continuous and categorical data, respectively. Several metrics were used to evaluate the models’ performance in identifying and classifying falls. These metrics included sensitivity, specificity, *F*_1_-score, positive predictive value (PPV), negative predictive value (NPV), accuracy, and area under the receiver operating characteristic curve (AUC-ROC). A weighted-averaging approach was used for multiclass classifications to report the overall model performance [34]. Furthermore, the percentage of the notes correctly classified for each task by machine learning and ICD approach were calculated and compared through chi-square test. A 0.05 type 1 error probability was considered significant.

### Ethical Considerations

The study protocol was approved by Mass General Brigham Institutional Review Board (number 2023P000741). The board waived participant consent due to the retrospective nature of the study. All the notes were de-identified in the preprocessing stage to avoid the inclusion of any protected health information (PHI) and to ensure patient privacy and compliance with HIPAA regulations.

## Results

**Figure 1.**
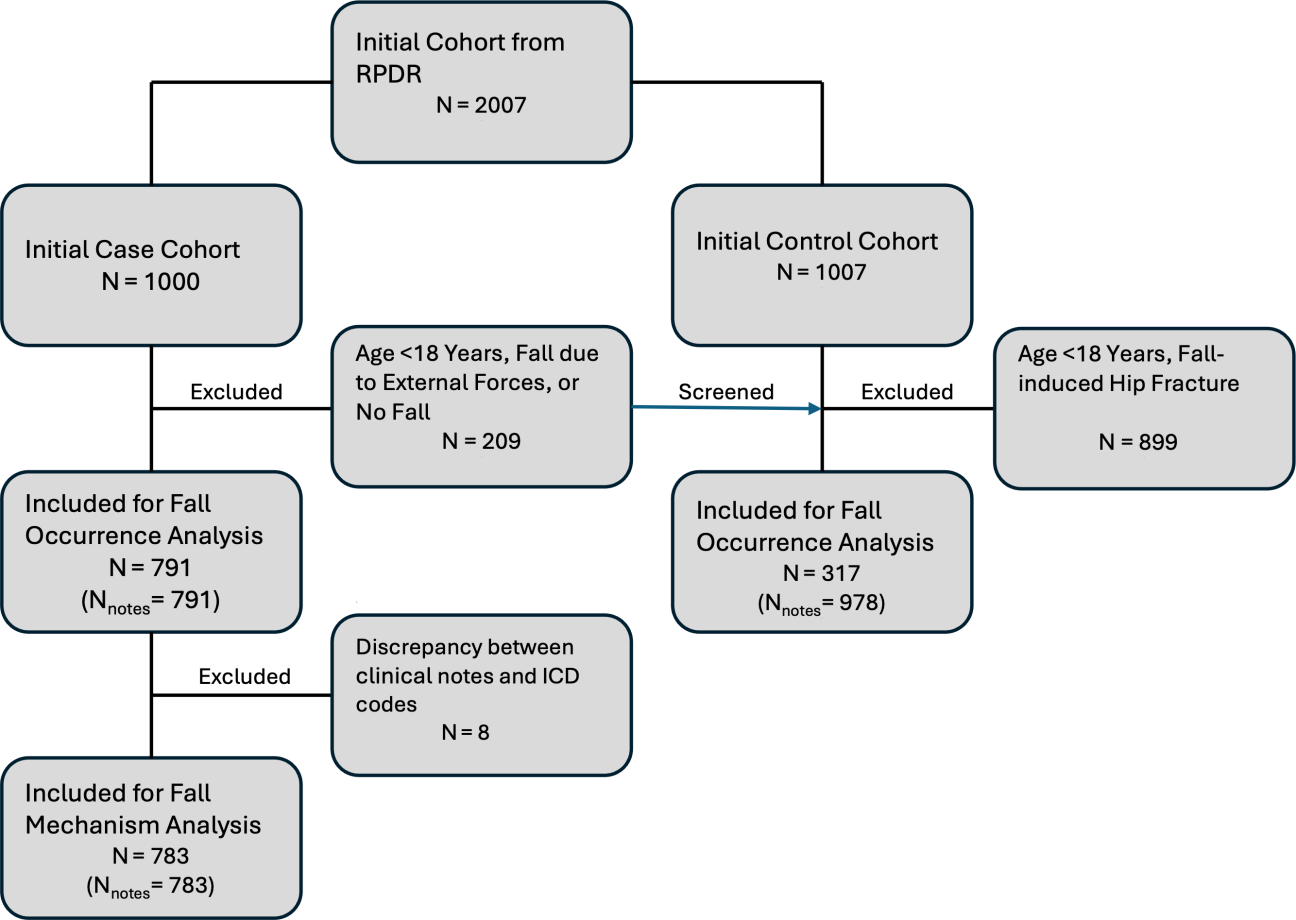
Study population flowchart. RPDR: Research Patient Data Registry.

A total of 1,769 clinical notes were analyzed for the fall occurrence task. Of these, 791 notes corresponded to the case group (one note per patient, n=791), and 978 notes were from the control group (representing 317 individuals with multiple notes per individual) (Figure 1). Moreover, for the fall mechanism classification task, 783 notes (one note per patient, n=783) were included, comprising 511 same-level falls, 151 multilevel falls, and 121 unclassified falls. The case group comprised older individuals with a mean age of 77.7 (SD 14.3) years versus 65.3 (SD 19.6) years of the control group (*P*<.001; Table 2). Furthermore, although both groups had a higher proportion of females, the case group had a notably higher percentage of female patients than the control group (*P*=.01; Table 2).

**Figure 2.**
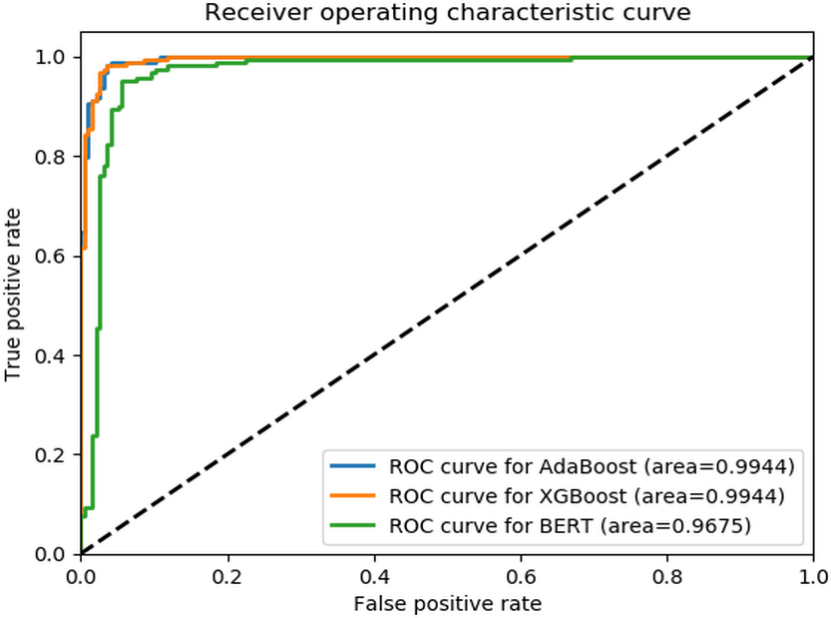
Receiver operating characteristic curve for the fall occurrence detection task. AdaBoost: adaptive boosting; BERT: Bidrectional Encoder Representations from Transformers; ROC: receiver operating characteristic; XGBoost: extreme gradient boosting.

All 3 models performed well for detecting fall occurrences, with the BERT model showing a lower *F*_1_-score and AUC-ROC (Table 3, Figure 2). The models could successfully classify a significant portion of patient notes (XGBoost=97%, AdaBoost=98%) as opposed to the *ICD* approach, which could find 26% of them (*P*<.001; Table 4).

**Table 2.** Comparison of the baseline characteristics of the study groups.

Group	Age (years), mean (SD)	Gender (female), n (%)	Race (White), n (%)
Fall (n=791)	77.7 (14.3)	520 (65.7)	700 (88.5)
No fall (n=317)	65.3 (19.6)	183 (57.7)	278 (87.7)
*P* value	<.001	.01	.61^a^

a Based on the comparison between the White and non-White races.

**Table 3.** The performance metrics of the study models for detection of fall occurrence and fall mechanism classification. Algorithms were trained on an expert annotated database.

Outcomes and models	PPV^a^	NPV^b^	Sensitivity	Specificity	*F*_1_-score	Accuracy	AUC-ROC^c^
Fall occurrence detection							
BERT^d^	0.94	0.88	0.84	0.96	0.88	0.90	0.97
AdaBoost^e^	0.95	0.98	0.98	0.96	0.97	0.97	0.99
XGBoost^f^	0.96	0.98	0.97	0.96	0.97	0.97	0.99
Fall mechanism classification^g^							
SVM^h^	0.56	0.50	0.62	0.36	0.57	0.62	0.67
AdaBoost	0.55	0.43	0.60	0.39	0.56	0.60	0.61
XGBoost	0.60	0.51	0.62	0.56	0.61	0.62	0.65
RF^i^	0.60	0.52	0.65	0.35	0.60	0.65	0.70

a PPV: positive predictive value.

b NPV: negative predictive value.

c AUC-ROC: area under the receiver operating characteristic curve.

d BERT: Bidirectional Encoder Representations from Transformers.

e AdaBoost: adaptive boosting.

f XGBoost: extreme gradient boosting.

g Weighted metrics are presented.

h SVM: support vector machine.

i RF: random forest.

**Table 4.** Percentage of fall notes correctly classified by natural language processing approach versus *International Classification of Diseases* codes.

Model	Fall occurrence	Fall mechanism			
		Overall	Class S^a^	Class M^b^	Class U^c^
*ICD* ^d^	26%	12%	8.4%	15.2%	22.2%
BERT^e^	84%	–^f^	–	–	–
AdaBoost^g^	98%	60%	82%	26.1%	11%
XGBoost^h^	97%	62%	78%	37%	28%
SVM^i^	–	62%	87%	17.4%	14%
RF^j^	–	65%	88.3%	15.2%	28%

a Class S: same-level.

b Class M: multi-level.

c Class U: unclassified.

d *ICD*: *International Classification of Diseases*.

e BERT: Bidirectional Encoder Representations from Transformers.

f Not available.

g AdaBoost: adaptive boosting.

h XGBoost: extreme gradient boosting

i SVM: support vector machine.

j RF: random forest.

**Figure 3.**
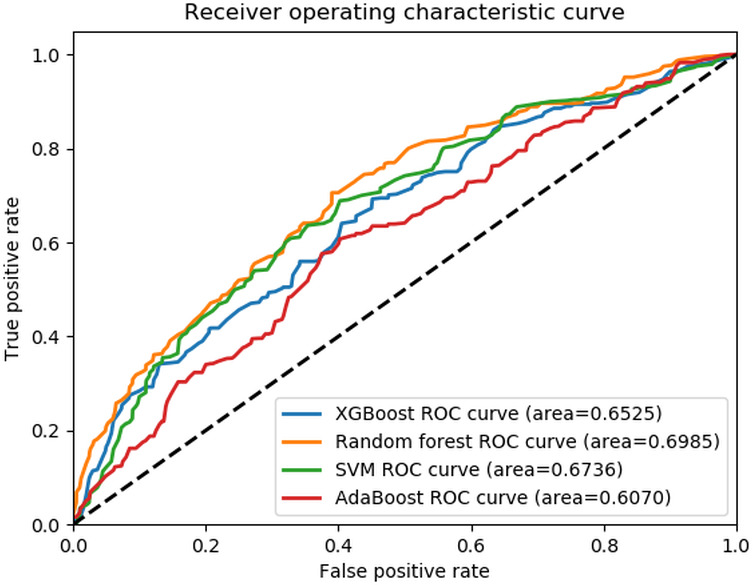
Receiver operating characteristics curve for the fall mechanism.AdaBoost: adaptive boosting; ROC: receiver operating characteristic; SVM: support vector machine; XGBoost: extreme gradient boosting.

Regarding fall mechanism classification, the RF model slightly outperformed the others with an AUC-ROC of 0.70 and an *F*_1_-score of 0.60 (Table 3, Figure 3). Moreover, the RF model correctly classified fall mechanism in 65% of the fall notes compared to the 12% of the *ICD* method (*P*<.001, Table 4.). However, all 4 NLP models showed high classification performance in identifying small-level class falls only (Table 4).

## Discussion

This study aimed to automate fall identification and classification based on its mechanism from clinical notes and subsequently compare the results with the traditional *ICD* approach for building fall registries. Our results demonstrated the superior performance of NLP models, which correctly identified 98% of the notes for fall occurrence compared to the 26% detected by the *ICD* approach. Furthermore, the models could classify 65% of fall mechanisms, while the ICD approach detected 12% of these cases.

Automated identification of fall incidents from clinical notes is an emerging topic in biomedical sciences. It serves multiple purposes, such as insurance claim processing, cost analysis for falls, and enhancing fall prevention measures for inpatient safety [35-37]. Despite these varied objectives, there are commonalities in the methodologies and models used. However, the interpretation of results can vary significantly and must be tailored to the specific study goals. Cheligeer et al [38] highlighted the superior performance of BERT and machine learning models in detecting inpatient falls compared to traditional *ICD* coding. Their findings underscored these models’ ability to accurately identify nonfall cases, as evidenced by high NPV and specificity. Nevertheless, when aiming to develop a comprehensive registry, achieving optimal sensitivity to maximize the inclusion of fall patients, alongside a high *F*_1_-score to balance PPV and sensitivity, becomes crucial.

Classical machine learning methods are commonly used in fall classification studies. Luther et al developed an SVM model using free-text clinical notes and a term-document matrix for feature selection, achieving an *F*_1_-score of 0.87 [39]. Our study extends this by using a TF-IDF feature selection method, which weighs terms based on their importance in capturing nuanced information from the notes. We found that ensemble methods achieved optimal performance with an *F*_1_-score of up to 0.98. Santos et al demonstrated superior performance of neural networks over classical machine learning methods [40]. This finding is supported by Fu et al, who showed high performance of context-aware models like BERT in fall detection tasks [17]. However, in our study, BERT did not outperform other machine learning models. BERT’s effectiveness depends on the availability of sufficient training data due to its deep learning architecture [41]. Therefore, the sample size in our study may have influenced the effectiveness of training within this framework.

Identifying fall mechanisms from patient records presents a significant challenge, which, if addressed properly, can provide invaluable information for clinical and quality improvement purposes. Roudsari et al investigated the acute cost of care for falls in patients over 65 years of age, categorized by *ICD* codes for mechanisms [13]. They found that same-level falls were the most common mechanism of injury (28%). However, most falls (60%) were coded as unspecified falls without mentioning the mechanism. In our study, only 11% of the notes were coded specifically for falls, and surprisingly, there were occasional discrepancies between the coded mechanisms and those described in clinical notes. Whether this discrepancy stems from insufficient clinical information or a tendency among providers to prioritize documenting immediate medical needs requires further investigation. Relying solely on medical coding is unreliable for identifying fall mechanisms.

While NLP has shown promise in retrieving data from medical records, its application in fall mechanism extraction remains underexplored. Liu et al automated the extraction of inpatient fall severity from incident reports, leveraging structured features to improve the *F*_1_-score by 8%, achieving 0.78 [22]. Our study incorporated diverse types of unstructured clinical notes, including discharge summaries and progress notes. These notes were authored by various medical professionals with differing styles and descriptions of falls, introducing significant variability that posed challenges for extracting features. Our results indicated that the XGBoost and RF models achieved the highest *F*_1_-scores (0.6). These findings are consistent with previous research demonstrating improved disease classification accuracy using ensemble methods applied to medical notes [22]. Additionally, using ensemble methods, Albano et al have shown promise in enhancing the classification accuracy when dealing with rare classes [42]. However, our study revealed suboptimal performance of the models in managing the “multilevel” and “unclassified” subclasses, likely due to the overall limited number of notes available for these classes. Although reflective of real-life scenarios, the imbalance in fall mechanism classes may have impacted the performance of the models. Ensemble models like XGBoost and RF are prone to overfitting patterns in the training data, especially when managing imbalanced datasets. Similarly, even after fine-tuning, BERT may carry over biases from its general-purpose pretraining, limiting its ability to capture domain-specific nuances in clinical notes fully. To address these challenges, we applied weighted evaluation metrics to ensure a fair performance assessment across all classes. Additionally, hyperparameters were systematically optimized to mitigate class imbalance, and BERT was fine-tuned explicitly on clinical notes to enhance its applicability to the domain. However, relying on weighted metrics and fine-tuning may not entirely overcome the inherent limitations of dataset imbalance and pretraining biases. Future work should focus on augmenting the dataset to improve class balance and explore alternative architectures or pretraining strategies to reduce bias and overfitting.

Different approaches can be adopted for planning health care registries based on the registry’s purpose, target population, and source data structure [43]. Administrative codes are commonly used to build retrospective registries when using health records. However, the accuracy of this method is not universally reliable across all medical conditions [44,45]. For example, a study by Dal et al evaluated the accuracy of the *ICD*-based Danish National Registry of Patients in identifying individuals with acromegaly, reporting a PPV of only 54.2% (CI 48.3‐60) compared to expert-confirmed diagnoses [46]. Similarly, *ICD* codes for falls are often inconsistently applied, making them an unreliable sole method for identifying fall incidents. Our results highlight the potential of automated clinical note screening using NLP as an alternative for building registries. However, NLP can be computationally intensive due to the broad scope of falls, which spans diverse patient populations and clinical scenarios. This study proposes a combined approach using administrative codes related to fall conditions as a prescreening step to narrow the dataset, followed by NLP-based automated screening of clinical notes. This strategy balances computational efficiency with improved accuracy in registry development. Furthermore, this approach offers the advantage of extracting additional clinical details, such as the fall mechanism, which are often unavailable in administrative codes but crucial for understanding and preventing falls [47].

Although this study made important strides in developing fall registries, there are a few areas for improvement. The sample size was adequate for a retrospective analysis; however, larger and more diverse populations would enhance the robustness of machine learning models. Additionally, our dataset was predominantly composed of individuals of White race, reflecting the demographic characteristics of the region. This provides an opportunity to expand the research to include more diverse groups. We also acknowledge recent advancements in data preprocessing, including automated entity resolution and noise handling, which can be used in future studies to enhance robustness and scalability [48]. To address these issues, our future efforts will be focused on external validation, incorporating broader and more representative populations to improve the generalizability and impact of the findings.

In conclusion, our findings demonstrated a promising performance of NLP methods in identifying patients with a history of falls and hip fractures and their fall mechanisms from clinical notes. This approach can significantly enhance the accuracy and efficiency of developing fall registries. Moreover, the models were particularly effective in classifying the mechanisms of falls in patients who experienced same-level falls. Future studies with larger sample sizes and a broader spectrum of pathologies can further validate these findings and address the class imbalance issue. If well-expanded and developed, our approach can be introduced to the health care systems as an efficient and cost-effective approach for developing valid and reliable registry systems of diseases or clinical conditions that greatly burden the health care systems and the patients.

## Supplementary material

10.2196/66973 Multimedia Appendix 1 Supplementary tables detailing an overview of the text segmentation process and model development.

## Abbreviations

AdaBoost: adaptive boosting
AUC-ROC: area under the receiver operating characteristic curve
BERT: Bidirectional Encoder Representations from Transformers
CPT: Current Procedural Terminology
*ICD*: *International Classification of Diseases*
NLP: natural language processing
NPV: negative predictive value
PPV: positive predictive value
RF: random forest
SVM: support vector machine
TF-IDF: Term Frequency-Inverse Document Frequency
XGBoost: extreme gradient boosting
