# Supplementary material for: Performance of Natural Language Processing versus International Classification of Diseases Codes in Building Registries for Patients With Fall Injury: Retrospective Analysis
Source: JMIR Med Inform. 2025 Jul 14;13:e66973. doi: 10.2196/66973 (PMC12279314; doi:10.2196/66973)
Supplement: Multimedia Appendix 1 [file medinform-v13-e66973-s001.docx]

**Table S1.** Overview of the text segmentation process.

| Condition | Subcondition 1 | Subcondition 2 | Example |
| --- | --- | --- | --- |
| Contains a word followed by colon | Segment contains no period in first 10 characters | First letter is capitalized | “Admission date:” |
|  | Contains a “.” in the first 10 characters (indicates a trail belonging to the previous section). | Part after “.” should contain at least 1 character. | “and the admitting diagnosis is shoulder pain. Check-up date: Saturday” (the text before the first period is added to the previous section, while the content after becomes a new section heading). |
| Consists of all uppercase character | First character is not a digit (exclude time-related information “7 AM”, dosages “500 MG PO”, and prescription list “1. VITAMIN D3” |  | “HISTORY & PRESENT ILLNESS” |

**Table S2.** This algorithm parses a block of text, segments it into sections based on predefined indicators, and stores these sections in a structured format.

| 1: Initialize an empty list to store the final list of complete sections.  2: Initialize the section head as None.  3: Split the text into parts based on newline characters (‘\n’).  4: Remove trailing white spaces and empty segments.  5: **for** each segment obtained from the split **do**  6: **if** segment contains indicators for being section head, **then**  7: **if** segment meets full conditions to be section head, **then**  8: Make segment section head  9: Set the body state to False  10: **else if** section head is not None **then**  11: Append the segment to the section head.  12: Set the body state to True.  13: **else**  14: Make segment section head  15: Set body to False  16: **end if**  17: **else**  18: **if** section head is None then ▷ # for initial segments without section head  19: Make segment section head  20: Set body to False  21: **else**  22: Append the segment to section head.  23: Set body to True  24: **end if**  25: **end if**  26: **if** body is False **then**  27: Append section head to complete list of sections  28: **end if**  29: **end for**  **return** Final list of complete sections. |
| --- |

**Table S3.** Hyperparameter values explored during grid search and the selected optimal values.

| Outcome | Model | Parameter | Explored values | Selected values |
| --- | --- | --- | --- | --- |
| Fall occurrence detection | AdaBoost | N_estimator | [100, 200, 300, 400] | 200 |
|  |  | Learning Rate | [0.1, 0.5, 1] | 1 |
|  |  | Max_depth | [1, 3, 5] | 5 |
|  | XGBoost | N_estimator | [100, 200, 300] | 200 |
|  |  | Learning Rate | [0.1, 0.2, 0.3] | 0.2 |
|  |  | Max_depth | [3, 5, 7] | 3 |
|  |  | Colsample_bytree | [0.5, 0.8, 1] | 0.5 |
| Fall mechanism classification | AdaBoost | N_estimator | [100,200, 400] | 200 |
|  |  | Learning Rate | [0.1, 0.5, 1] | 1 |
|  |  | Max_depth | [1, 3, 5] | 5 |
|  | XGBoost | N_estimator | [100, 300] | 100 |
|  |  | Learning Rate | [0.2, 0.3, 0.5] | 0.3 |
|  |  | Max_depth | [3, 5, 7] | 5 |
|  |  | Colsample_bytree | [0.5, 0.8, 1] | 0.5 |
|  | SVM | C | [1, 10, 100] | 10 |
|  |  | Kernel | [Linear, RBF] | RBF |
|  |  | Gamma | [Scale, Auto] | Scale |
|  | RF | N_estimator | [100, 200, 300] | 200 |
|  |  | Max_depth | [10, 20, 30] | 30 |
|  |  | Min_samples_split | [2, 5, 10] | 10 |
|  |  | Min_samples_leaf | [1, 2, 4] | 1 |
